# Editorial: Targeting the aging mitochondria: mechanisms, methods, and therapeutic strategies

**DOI:** 10.3389/fnagi.2025.1591288

**Published:** 2025-03-27

**Authors:** Zhiquan Li, Konstantinos Palikaras, Yuan Li, Vilhelm A. Bohr

**Affiliations:** ^1^Department of Cellular and Molecular Medicine, Faculty of Health and Medical Sciences, University of Copenhagen, Copenhagen, Denmark; ^2^Department of Physiology, Medical School, National and Kapodistrian University of Athens, Athens, Greece

**Keywords:** mitochondrial dysfunction, synapse, cuproptosis, PGC-1α, FOXO, oxidative stress, neurodegeneration, reproductive aging

**Figure 1:**
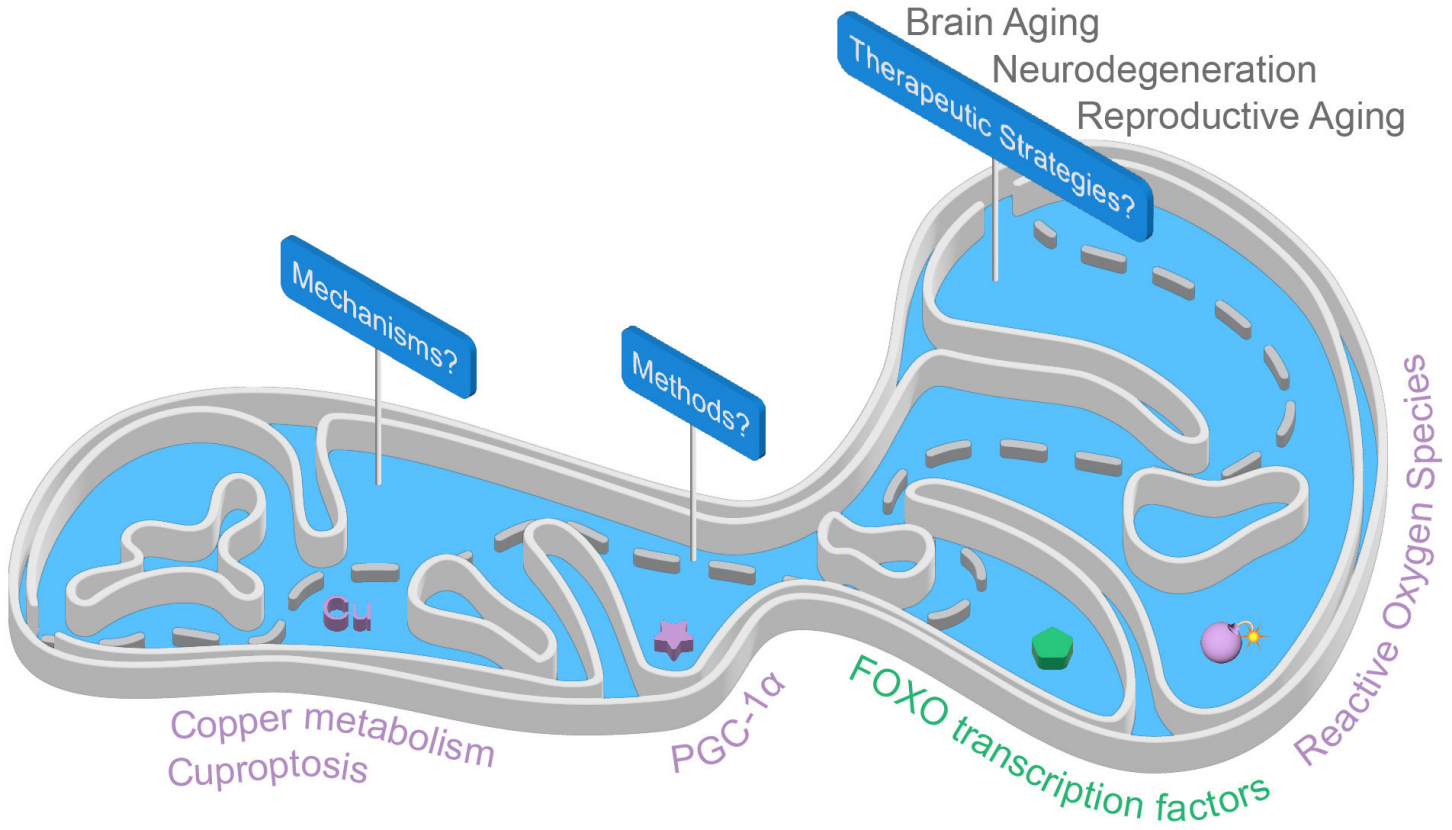
The mitochondrial path: from mechanisms to therapeutics. Several mitochondria-targeted approaches show promise for promoting healthy brain aging, combating neurodegeneration, and addressing reproductive aging. This Research Topic highlights a new form of mitochondria-associated programmed cell death called cuproptosis, the master regulator of mitochondrial biogenesis PGC-1α, and oxidative stress responses where FOXO transcription factors play a pivotal role in mitochondrial homeostasis.

Aging remains one of the greatest challenges in modern medicine, impacting several aspects of human health, from frailty and cognitive resilience to reproductive aging. The intricate mechanisms that govern aging are coming into sharper focus, uncovering new opportunities for potential interventions. Four recent reviews provide compelling insights into the molecular and cellular pathways underlying neurological and reproductive aging. By focusing on mitochondria, they highlight how targeting mitochondrial function, synaptic plasticity, copper metabolism, and oxidative stress could pave the way for innovative therapeutic strategies (Figure 1).

Among the well-recognized hallmarks of aging (Schmauck-Medina et al., 2022), genomic instability (Li et al., 2023), mitochondrial dysfunction (Hou et al., 2019; Li et al., 2024), and nuclear-mitochondria communications are crucial for the maintenance of cellular and organismal health. Mitochondria, found in nearly all cell types, and especially in high energy-demanding neurons, play a fundamental role in energy production, metabolic regulation, redox homeostasis, and cell fate determination. Notably, they can be transferred between cells and even inherited across generations, highlighting their pivotal role in maintaining physiological function and longevity.

Synaptic plasticity is fundamental for preserving brain function. Mitochondrial deficits, particularly those affecting organelle distribution, motility, and calcium buffering capacity, have been associated with impairments in synaptic transmission, potentiation, and plasticity (Todorova and Blokland, 2017). Navakkode and Kennedy highlight the importance of assessing cognition alongside physical fitness and lifespan in healthy aging studies. Importantly, cognitive decline is not an inevitable consequence of aging but rather a process shaped by genetic, environmental, and lifestyle factors. Maintaining brain functions and cognitive reserves by identifying key molecular drivers of synaptic resilience and enhancing neuroplasticity (possibly through mitochondria-targeted interventions) could provide a proactive strategy for preserving cognitive function in aging populations.

In neurological health, mitochondria also play a role in programmed cell death, like apoptosis, mitophagy-associated cell death, pyroptosis, necroptosis, ferroptosis, and cuproptosis (Zhu et al., 2024). Cuproptosis has been identified as a novel form of copper-dependent and mitochondria-mediated programmed cell death (Tsvetkov et al., 2022; Wang et al., 2022). Fan et al. emphasize that this emerging concept adds a new dimension to understanding age-related diseases. While copper is essential for enzymatic functions and cellular homeostasis, its dysregulation can lead to protein aggregation and cytotoxicity, exacerbating neurodegeneration and systemic aging. In the future, understanding the role of mitochondria in cuproptosis could provide potential therapeutic targets for mitigating age-related pathologies, underscoring the importance of precision medicine in aging research.

Mitochondrial functions are largely coordinated by the master regulator peroxisome proliferator-activated receptor-γ coactivator-1α (PGC-1α), and its dysregulation is a common feature in many neurological diseases (Panes et al., 2022). Its role in neurological diseases highlights the importance of mitochondrial biosynthesis and metabolic regulation in the maintenance of neuronal integrity. Tang et al. describe PGC-1α as a protective factor against neuroinflammation, oxidative stress, and autophagy-related dysfunction in conditions such as Alzheimer's disease, Parkinson's disease, and amyotrophic lateral sclerosis. They also discuss various compounds and drug-targeting strategies that may ameliorate these pathologies. As our understanding deepens, targeting PGC-1α could offer promising therapeutic avenues for improving mitochondrial health and slowing or even preventing neurodegeneration. Future studies should focus on optimizing PGC-1α modulation to enhance its protective effects while minimizing potential off-target consequences.

The regulatory hub of FOXO transcription factors and PGC-1α plays a pivotal role in protecting cells from oxidative stress. Mitochondrial dysfunction and excessive reactive oxygen species (ROS) generation are closely linked to neurodegeneration and reproductive aging. Notably, a recent study also emphasizes FOXO by identifying its new target, OSER1, which helps to withstand oxidative stress and is associated with reproductive aging (Song et al., 2024). Song et al. underscore the critical role of redox balance in maintaining germ cell health and fertility. Given mitochondria's fundamental role in energy production and cellular homeostasis, targeting mitochondrial pathways to reduce oxidative stress could offer promising strategies to extend the reproductive lifespan in both men and women. The interplay between oxidative stress and mitochondrial damage creates a cycle that accelerates age-related declines in reproductive function. Understanding these mechanisms is crucial for developing effective therapies to address age-related infertility. The intersection of mitochondrial health and reproductive longevity remains a compelling frontier in aging-related research.

The convergence of these studies highlights the multifaceted nature of aging and the need for a holistic approach to its mitigation. Mitochondrial integrity, oxidative stress regulation, metal ion homeostasis, and synaptic adaptability form interconnected pathways that influence aging at multiple levels. By integrating insights from neurology, reproductive biology, and molecular aging research, scientists and clinicians can develop more effective mitochondrial-targeted interventions to enhance both lifespan and healthspan.

As we continue to unravel the molecular mechanisms of aging, targeting mitochondria presents a promising strategy for delaying or even preventing neurodegenerative diseases, minimizing cognitive decline, and preserving reproductive health. The challenge now lies in translating these groundbreaking discoveries into tangible therapies to promote longevity and healthy aging.
